# Increased Dementia Mortality in West Virginia Counties with Mountaintop Removal Mining?

**DOI:** 10.3390/ijerph16214278

**Published:** 2019-11-04

**Authors:** A. K. Salm, Michael J. Benson

**Affiliations:** 1 Department of Neurobiology and Anatomy, West Virginia University School of Medicine, Morgantown, WV 26506, USA; mjbenson@mix.wvu.edu; 2 Department of Pathology, Anatomy and Laboratory Medicine, West Virginia University School of Medicine, Morgantown, WV 26506, USA

**Keywords:** mountaintop removal mining, dementia mortality, Alzheimer’s disease mortality, West Virginia

## Abstract

Atmospheric particulate matter (PM) is elevated in areas of mountaintop removal mining (MTM), a practice that has been ongoing in some counties of West Virginia (WV) USA since the 1970s. PM inhalation has been linked to central nervous system pathophysiology, including cognitive decline and dementia. Here we compared county dementia mortality statistics in MTM vs. non-MTM WV counties over a period spanning 2001–2015. We found significantly elevated age-adjusted vascular or unspecified dementia mortality/100,000 population in WV MTM counties where, after adjusting for socioeconomic variables, dementia mortality was 15.60 (±3.14 Standard Error of the Mean (S.E.M.)) times higher than that of non-MTM counties. Further analyses with satellite imaging data revealed a highly significant positive correlation between the number of *distinct mining sites* vs. both mean and cumulative vascular and unspecified dementia mortality over the 15 year period. This was in contrast to finding only a weak relationship between dementia mortality rates and the *overall square kilometers* mined. No effect of living in an MTM county was found for the rate of Alzheimer’s type dementia and possible reasons for this are considered. Based on these results, and the current literature, we hypothesize that inhalation of PM associated with MTM contributes to dementia mortality of the vascular or unspecified types. However, limitations inherent in ecological-type studies such as this, preclude definitive extrapolation to individuals in MTM-counties at this time. We hope these findings will inspire follow-up cohort and case-controlled type studies to determine if specific causative factors associated with living near MTM can be identified. Given the need for caregiving and medical support, increased dementia mortality of the magnitude seen here could, unfortunately, place great demands upon MTM county public health resources in the future.

## 1. Introduction

Surface coal mining has been ongoing in Central Appalachia for well over a century and continues still. Mountaintop removal mining (MTM) is an increasingly prevalent method of surface coal mining dependent upon the explosive removal of mountaintops to reveal and extract the coal seams underlying the earth’s surface. It has become widespread in the Central Appalachian region of the eastern U.S. since the 1970s. MTM has been associated with increased birth defects [1], cardiovascular disease [2], self-reported cancer rates [3] and days of poor physical and mental health [4]. By nature, such community health studies have limitations with respect to exposure characterization (see [5] for review), and further targeted studies are yet needed to determine whether and how environmental factors might contribute to these findings. Nevertheless, a National Academy of Sciences and Engineering study of possible health risks posed to individuals living near MTM sites in Central Appalachia was suspended in 2017 by the U.S. Department of the Interior, citing budgetary constraints and industry assertions that MTM is in decline [6,7,8]. The study panel was ultimately dismissed [9].

Exposure to poor air quality is a frequent occurrence for those living near MTM sites as it creates coarse (>2.5 m), fine (≤2.5 m) and ultrafine (<0.1 m) airborne particulate matter (PM) [10]. Combusted explosives used in the mining process also release ammonium nitrate PM, as well as gases such as carbon dioxide, carbon monoxide, nitric oxide, sulfur dioxide and ammonia [11,12] as cited by [13]. Periodic samples taken over multiple days indicate disruption of the overburden of soil and minerals, which can result in airborne concentrations of crustal elements nearly six times higher than in non-mining areas [13]. Vehicular traffic on mining haul roads, often traversing populated valleys, also contributes dust and diesel emissions to the mix [14]. Air monitoring studies in MTM counties have shown atmospheric concentrations of PM comparable to that of urban areas where PM exposure has been implicated in elevated rates of respiratory and cardiovascular disease [10,15,16]. Satellite-based observations of PM levels in the vicinity of MTM operations have shown that elevated coal and vehicular traffic dust can be chronic, i.e., consistently evident when multiple measures were taken over the course of a year [17].

With respect to central nervous system (CNS) effects, that PM gains direct access to nervous system components is now widely accepted [18]. Exposure to PM_2.5_ in particular has been associated with increased hospital admissions for dementia, Alzheimer’s disease (AD) and Parkinson’s disease [19,20]. Self-reports link living near MTM sites with diverse neurological signs such as dizziness, fainting, headaches, tremors and seizures [21]. Ultrafine PM has been found to be particularly detrimental to brain health [22,23] and epidemiological work supports a possible relationship between high urban ultrafine PM and cognitive decline, neurodegeneration and dementia [19,24,25,26,27,28]. The cellular mechanisms by which PM gains access to the relatively well-protected CNS are still being elucidated. However, two routes across the blood–brain barrier appear to be via the olfactory [22,23] and trigeminal [23] neural pathways which extend from the nasal cavity into the brain. PM-associated cellular activation and neuroinflammation (associated with neurodegenerative diseases) has been documented [29,30,31] along these neural projections.

Together, the PM generated by MTM and the emerging case for a causative link between PM and neurodegenerative diseases motivated us to conduct this preliminary ecological analysis [32] to determine if differences in dementia mortality exist between West Virginia counties where MTM does, and does not, occur.

## 2. Materials and Methods

### 2.1. Public Health and Socioeconomic Data

The West Virginia Department of Health and Human Resources (DHHR) provided underlying cause of death statistics based on death certificate data filed in the state office of vital statistics for the years 2001–2015. Provided data for each year included: county population, number of vascular and unspecified dementia deaths (ICD codes F01, F03) and Alzheimer’s dementia (AD; ICD code G30) deaths, the crude rate of dementia and AD deaths per 100,000 population and the age-adjusted rate of dementia and AD deaths per 100,000 population. The WV DHHR also supplied us with national average dementia data (means and Standard Errors of the Mean (S.E.M.s) only) which had been downloaded from the Center for Disease Control Wonder website, https://wonder.cdc.gov, on 17 November 2017. All data were de-identified. Underlying cause of death designations on West Virginia death certificates are based on the Center for Disease Control and Prevention’s *International Statistical Classification of Diseases and Related Health Problems, Tenth Revision* (ICD 10) [33]. Note that WV data obtained from the WV DHHR are somewhat more updated than that available from CDC WONDER in that the former closes its files to new death certificate data somewhat later than does the CDC. However, these differences are minor where they exist (D. Christy, WV DHHR, personal communication).

Since an increased risk of cardiovascular disease (CVD) in populations living in the vicinity of MTM has been previously reported [2] and cardiovascular disease is a significant risk factor for dementia [34,35], we also evaluated patterns of CVD vs. dementia mortality in mining vs. non-mining counties over the same 2001–2015 time span. Data concerning the age-adjusted mortality/100,000 population for *Major Cardiovascular Diseases (I00-I78)* for all West Virginia counties covering the years 2001–2015 were downloaded (4 April 2018) from the Center for Disease Control and Prevention CDC WONDER database [36]. No further adjustments to these data were made.

Socioeconomic factors such as income and level of formal education have also been linked to increased risk for dementia mortality [37,38]. Therefore, county level mean household income as well as the percent of families below the poverty level were derived from the 5 year American Community Survey for 2005–2009 and 2010–2014 databases (https://factfinder.census.gov, downloaded 20 November 2018 and 14 October 2019). The poverty threshold for a family of four ranged from $19,971–24,230 over the 10 year span). Mean education levels for each county were similarly derived and averaged over the 10 year period from the from the 5 year American Community Survey for 2005–2009 and 2010–2014 databases (https://factfinder.census.gov, downloaded 20 November 2018). We chose to evaluate the percent of individuals 24 years of age or older within each county who had achieved a high school diploma (or equivalent) and the percentage of individuals in MTM vs. non-MTM counties aged 24 or older with a bachelor’s degree or higher. These databases together provided the longest period of non-overlapping and homogeneously organized data for all 55 counties that fell within the time-frame of our 2001–2015 dementia mortality data. (This period was ultimately found to correspond to that time where the steepest rise dementia mortality was seen). Data for each of the 54 counties were averaged over the 10 year period. 

### 2.2. Location and Extent of MTM in West Virginia

**Figure 1:**
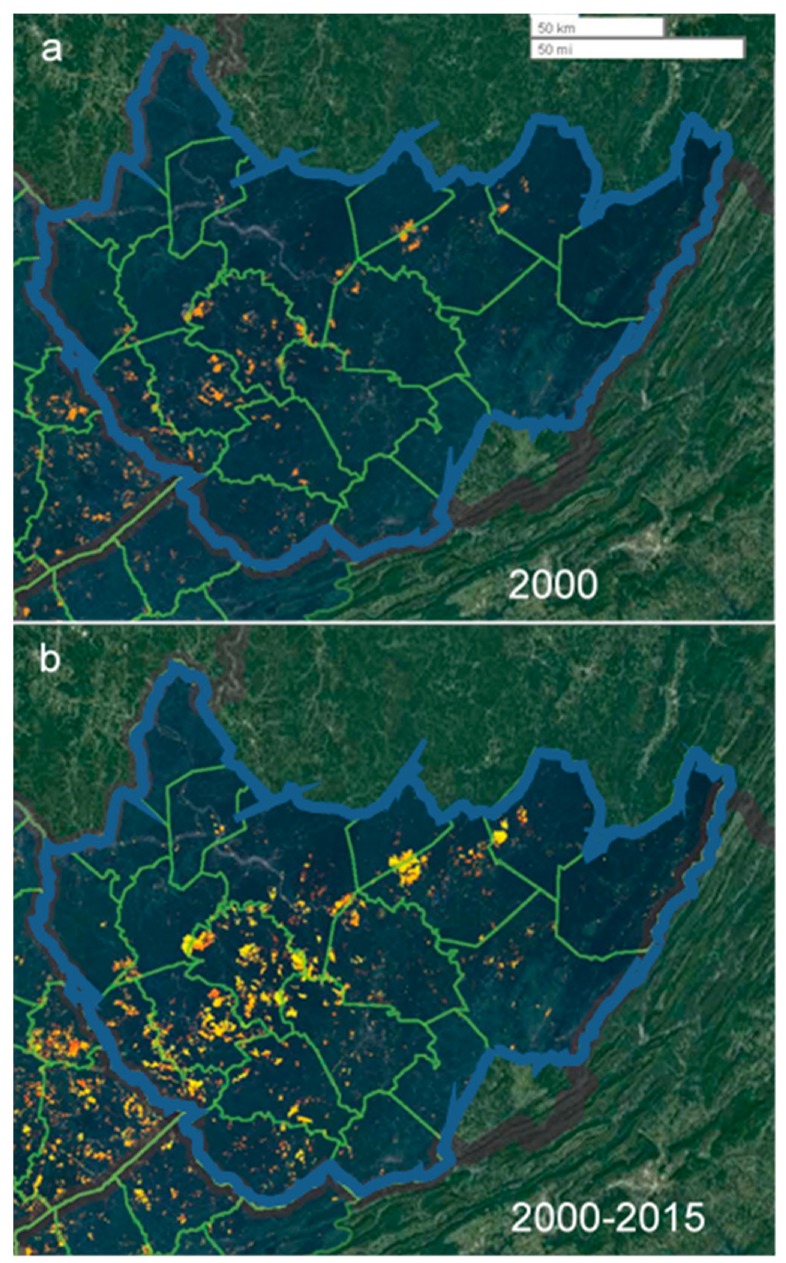
Surface mining in Southern West Virginia 2000–2015. Composite satellite and data analysis overlay images captured from the SkyTruth website, which depict the location and extent of mountaintop mining (MTM) operations in central Appalachia from 1985 to 2015. The 19 Southern West Virginia counties that were studied here are outlined in blue. Individual counties are outlined in green. We have added the overlay of dates to illustrate which years we have captured. (**a**) Location and extent of central Appalachian MTM as seen in the year 2000 and (**b**) 2000–2015 (cumulative). Hotter (redder) colors indicate data that are more recent. Scale bars = 50 km (top) and 50 miles (bottom) respectively. Source: SkyTruth mountaintop mining (MTM) http://Skytruthmtr.appspot.com/#closecontent. Map data: Google, Imagery ©2019 TerraMetrics, Leaflet | Stamen, ©OpenStreetMap, ©CartoDB, SkyTruth, © CARTO. This interactive website can be accessed at the above hyperlink where year by year changes in the location and extent of MTM can be viewed. Details of SkyTruth’s image acquisition and analyses determining the extent of MTM are detailed in [39].

Data from satellite imagery concerning the location and extent of MTM in WV counties were provided by SkyTruth (Figure 1). SkyTruth is a nonprofit organization that uses remote sensing and digital mapping to reveal and monitor an array of environmental parameters around the globe. To monitor MTM, Landsat (NASA/USGS) satellite imagery was used to accurately map the locations and yearly extent of MTM for 74 counties in eastern Kentucky, southern West Virginia, southwestern Virginia and eastern Tennessee. For this report, satellite data spanned the period of 2001–2015 and were confined to West Virginia where MTM is concentrated in the southern part of the state. The detailed methods and algorithms underlying SkyTruth’s data acquisition and analyses are described in [39]. In brief, three steps were followed that ensured that data acquisition and analysis were accurate. First, the Landsat images were obtained in a systematic fashion from a separate U.S. government agency, i.e., (NASA/USGS). Second, the image analysis process was automated—acres mined were derived from an algorithm which was systematically (blindly) applied to all images. Third, in a separate process, the authors verified the accuracy of mine site identification to be 92%. In addition to these factors, we also randomly spot checked their data against both the Google Earth dataset and the EIA.gov (Energy Information Administration, USA) yearly reports of surface mining.

**Figure 2:**
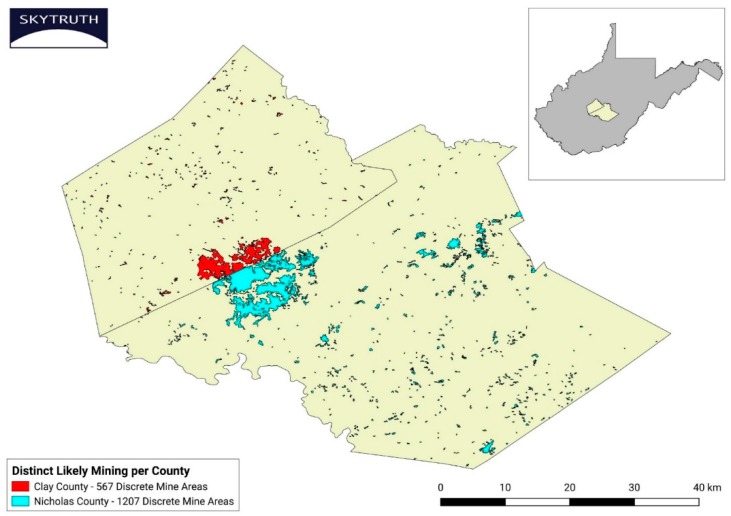
Distinct mining sites vs. km^2^ mined. An illustration, courtesy of SkyTruth, demonstrating “km^2^ mined” vs. “distinct mining sites” in Nicholas and Clay counties from 2000–2015. These composite images were created from multiple individual Landsat satellite images. As can be surmised from this composite, it is possible to have relatively little km^2^ mined but still have a great number of individual mines and vice versa. The number of individuals ultimately exposed to particulate matter (PM) from MTM operations would vary depending on the location and extent of mining in that county and the distribution of populated areas.

Yearly SkyTruth data for each county included (1) the number of distinct active mining sites; (2) the total square kilometers (km^2^) of active acres mined for that year, both of which were based on the unique spectral characteristics of vegetated forest vs. non-vegetated land; and (3) the cumulative km^2^ mined for that county. Active mining was defined as “any land where mining activity (i.e., earth removal and replacement) was likely occurring, or where mining activities had recently ceased so that the landscape still resembled a mine in active development”. Any individual Landsat image PIXEL (picture element) had to be classified as a mine site for all 365 days of a particular year to be counted as a mine for that year. Sites identified as an active mine in one year, would not be added again to the cumulative mining area total in future years such that “cumulative mining” was defined as “the non-duplicative summation of active mine area over time”. “Distinct likely mining sites” (Figure 2) were defined as “discrete areas of detection”. When a distinct site overlapped county boundaries, it was counted as a distinct site in each county. It should be noted that such distinct sites, as determined by satellite imagery, do not necessarily correspond to WV state, or mining company, mine boundary designations. It should also be noted that these methods do not distinguish between smaller surface mines vs. larger operations typically designated as “mountaintop removal mining”. However, as discussed in Pericak et al. [39], Appalachian coal is increasingly difficult to extract and now requires a greater area of active mining to obtain a metric ton of coal. Three times more area of mountainside per metric ton of coal was mined in 2015 vs. that required in the early 1990s [39]. Thus, the mining activity documented in our 2000–2015 time frame was increasingly likely to be classified as the mountaintop removal mining type, and no further efforts were made to differentiate between the two types of surface mining here.

To further corroborate the satellite imaging data, the short tons of surface mining coal production per county/per year, as reported on the EIA.gov website for the years 2001–2015, were randomly spot checked to confirm the presence of surface mining for the year. We also consulted the Google Earth compatible Map and GIS resources tool supplied by iLoveMountains.org. to further confirm every county designated by SkyTruth as having some amount of MTM activity within its boundaries for the year 2012 (“2012 extent of Mining Survey”).

### 2.3. Statistical Analyses

Except where noted, all data analyzed were age-adjusted per 100,000 population. GraphPad Prism version 7.0 for Mac or version 8 for PCs, (GraphPad Software Inc., La Jolla, CA, USA; www.graphpad.com) was used to carry out 2-way ANOVAs, Mann–Whitney and Student’s t-tests and Pearson’s r correlations. This statistical software assesses each data set to ensure that parametric assumptions of normality and homogeneous variance are met prior to performing parametric tests. Except where noted, the data analyzed here met such assumptions. SAS software (version 9.4; SAS Institute Inc., Cary, NC, USA) was used for the negative binomial regression and ANCOVA analyses.

Data availability statement: data used for this study are publically available from https://wonder.cdc.gov/, https://factfinder.census.gov, and via contacting the SkyTruth organization at https://www.skytruth.org/contact/ or from the corresponding author.

Spot checks of EIA.gov confirmed the presence and magnitude of surface mining as estimated from SkyTruth in the counties of interest. Since we had no a priori reason to exclude any particular county, and to avoid inadvertent biasing of the results, all 55 West Virginia counties were initially included in the analysis. However, with respect to Webster County, we observed a considerable surface mining operation as listed on the EIA.gov site. Since it was not included in the Skytruth dataset, it was ultimately decided to omit it from analysis. Thus, 19 counties which are situated adjacently in the southern part of the state were designated as MTM counties (surrounded by the blue line in Figure 1) and the remaining 35 were designated as non-MTM counties. Table 1 lists each county that was ultimately designated as having MTM over the 15 year period of the study, along with the minimum, maximum, mean and cumulative number of distinct sites, and the cumulative dementia mortality per 100,000 population. 

## 3. Results

In the years spanning 2001–2015, the number of distinct sites across the nineteen counties grew from 14,987 to 25,503. Within any one county, the number of distinct sites varied by as little as 147 sites (McDowell) to as many as 2579 sites (Raleigh County) across the 15 year period (Table 1).

**Figure 3:**
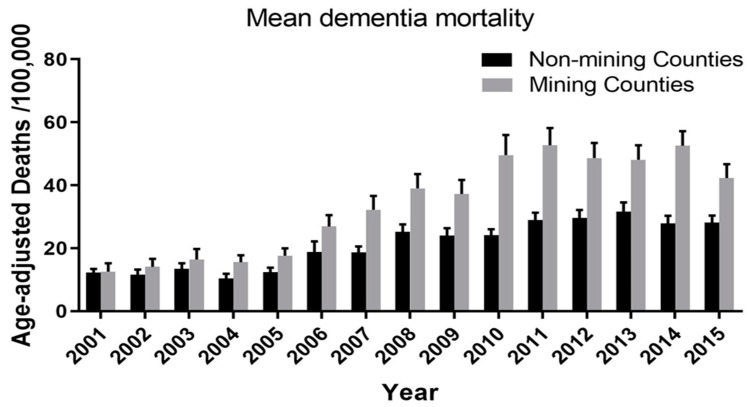
Age-adjusted vascular and unspecified dementia mortality/100,000 population in mining vs. non-mining counties in West Virginia over the years spanning 2000–2015. A 2-way repeated measures ANOVA was used to compare age-adjusted dementia mortality/100,000 population. An overall significant impact of mining on dementia mortality was found (F = 23.16; df = 1, 52; *p* < 0.0001). There was also a significant interaction between year and mining (F = 5.52; df = 14, 728; *p* < 0.0001) and a significant overall effect of year (F = 41.22; df = 14, 728; *p* < 0.0001).

*Dementia mortality: Mining* vs. *non-mining counties* (Figure 3): A 2-way repeated measures ANOVA was used to compare age-adjusted vascular and unspecified dementia mortality per 100,000 population in MTM vs. non-MTM counties over the 15 years spanning 2001–2015. An overall significant association was found between living in an MTM-county and increased dementia mortality (F = 23.16; df = 1, 52; *p* < 0.0001), i.e., on average there was more dementia mortality of this type in MTM counties over the 15 year period. There was also a significant interaction between year and county mining status: although dementia mortality increased over the 15 year period in both MTM and non-MTM counties, the increase was significantly greater in the MTM counties (F = 5.52; df = 14, 728; *p* < 0.0001; and see the negative binomial regression below). Last, there was also an overall effect of year (F = 41.22; df = 14; 728; *p* < 0.0001), reflecting that dementia mortality increased in *both* MTM and non-MTM counties over the 15 year period studied.

**Figure 4:**
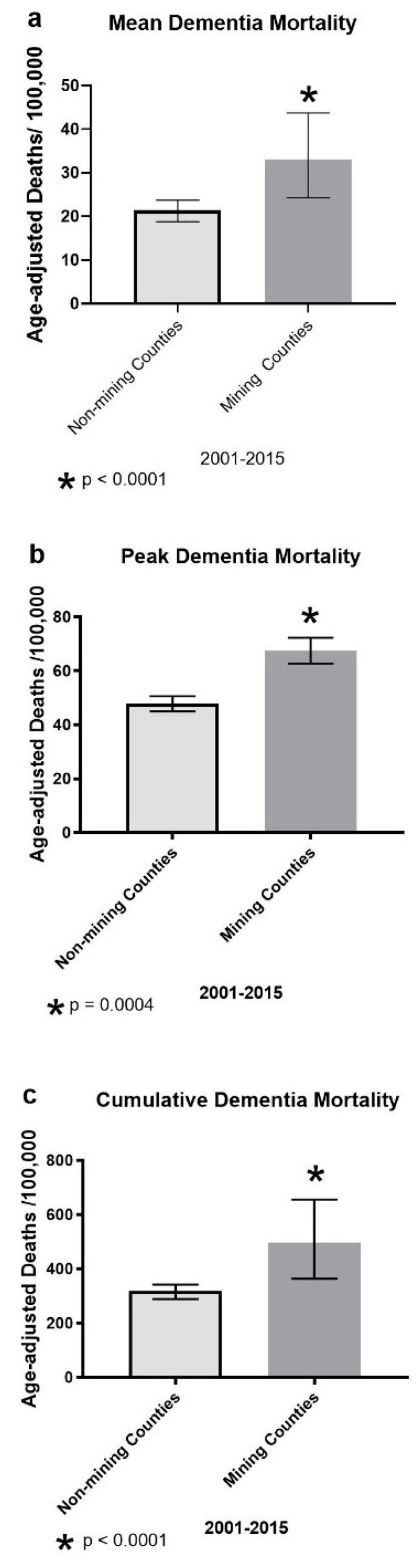
Mean, peak and cumulative age-adjusted vascular and unspecified dementia mortalities/100,000 population in non-mining vs. mining WV counties over the years spanning 2000–2015. All *p*-values are two-tailed. (**a**) The median mean dementia mortality in mining vs. non-mining counties in West Virginia. A Mann–Whitney U test for non-parametric distributions showed this measure was significantly higher in the mining counties (U = 119). The (median) mean deaths per year from dementia for mining vs. non-mining counties over the 15 year span was respectively 33.07 vs. 21.41 age-adjusted deaths per 100,000 population (Each plotted with a 95% C.I.: ±5.71, ±18.75). * denotes significantly different from non-mining counties, *p* < 0.0001). (**b**) Mean peak dementia mortality in mining vs. non-mining counties in West Virginia over the years spanning 2000–2015. An unpaired t-test found significantly higher peak dementia mortality in the mining counties (t = 3.77; df = 52; *p* = 0.0004). Mining counties had a mean (±SEM) peak value of 67.5 ± 4.81 age-adjusted deaths per 100,000 populations vs. 47.83 ± 2.82 for the non-mining counties. * denotes significantly different from non-mining counties; *p* = 0.0004). (**c**) As for cumulative dementia, a Mann–Whitney U test (again these data were non-parametric) showed significantly higher cumulative dementia mortality in MTM counties (4C; U = 121; *p* < 0.0001). The median cumulative death rates from dementia were 496 vs. 318 per 100,000 (Each plotted with a 95% C.I.: ±87.5, ±279.7).

To assess the relative magnitude of the differences in vascular and unspecified dementia mortality in MTM vs. non-MTM counties, we identified the mean, peak and cumulative rates of these dementias for each county across the 15 year span (Figure 4; all *p*-values are two-tailed). A Mann–Whitney U test (for non-parametric data) showed *mean* deaths from dementia were significantly higher in the MTM counties (Figure 4a; U = 119; *p* < 0.0001). The median (reported as such because these data were non-parametric) mean deaths per year from dementia for MTM vs. non-MTM counties over the 15 year span were respectively 33.07 vs. 21.41 age-adjusted deaths per 100,000 (Each plotted with a 95% C.I.: ±5.71, ±18.75).

An unpaired t-test found significantly higher *peak* dementia mortality in the MTM counties (Figure 4b; t = 3.77; df = 52; *p* = 0.0004). MTM counties had a mean (±SEM) peak value of 67.5 ± 4.81 vs. 47.83 ± 2.82 for the non-MTM counties. 

As for the 15 year *cumulative* dementia, a Mann–Whitney U test (for non-parametric data) showed significantly higher cumulative dementia mortality in MTM counties (Figure 4c; U = 121; *p* < 0.0001). The median values for cumulative death rates from dementia over the 15 year period for mining vs. non-mining counties were respectively 496 vs. 318 per 100,000 (Each plotted with a 95% C.I.: ±87.5, ±279.7). for these comparisons.

**Figure 5:**
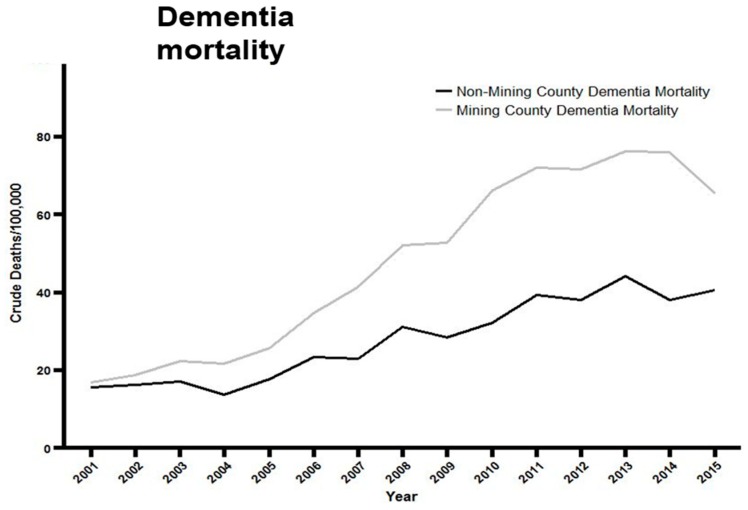
A negative binomial regression with a log link function was used to compare the relative trajectories of the increases in vascular and unspecified dementia mortality in mining vs. non-mining counties. The interaction between year and mining counties was significant (*p* = 0.007). On a year by year basis, the rate of dementia mortality increased 12.7% each year in mining counties compared to 8.9% in non-mining counties. The Incident Rate Ratio for this interaction is 1.035, indicating that for every 1 year increase in time, the rate of dementia mortality in mining counties increases 3.5% (95% C.I.: 1.0%–6.2%) more in mining counties than the rate of dementia mortality in non-mining counties.

To confirm and expand our interpretations of the 2-way ANOVA results (Figure 3 above) an analysis using SAS software (version 9.4; SAS Institute Inc., Cary, NC, USA), compared the relative trajectories of vascular and unspecified dementia mortality increases in MTM vs. non-MTM counties across the 15 year span. Due to the nature of the data set, which was not stratified according to age, crude (unadjusted) dementia mortality rates by county mining status were plotted across time (Figure 5). A negative binomial regression with a log link function was used to compare linear trends. Time was modeled as a continuous variable, non-MTM counties served as the reference group for county mining status and an interaction term between county mining status and time was incorporated. As seen in the initial 2-way ANOVA described above (Figure 3), the interaction between year and county mining status was again significant (*p* = 0.007). The Incident Rate Ratio (IRR) for this interaction was 1.035, which indicates that for every 1 year increase in time, the rate of dementia mortality in MTM counties increased 3.5% (95% C.I.: 1.0%–6.2%) more than that of the non-MTM counties. On a year by year basis, the rate of dementia mortality increased 12.7% each year in mining counties compared to an increase of 8.9% in non-mining counties. 

**Figure 6:**
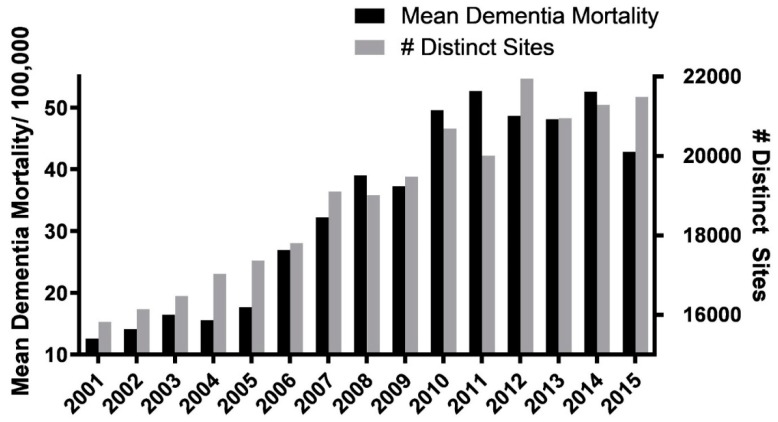
Distinct sites vs. mean mortality. Graphic illustration of nearly identical trajectories of increase in the numbers of distinct MTM sites (total sites) vs. the mean age-adjusted mining county vascular and unspecified dementia mortality rates/100,000 population between 2001 and 2015.

When we examined the *number of distinct mining sites per year,* we found nearly identical trajectories of increase over the 15 year period for this variable vs. the *mean mining county dementia mortality rates* (Figure 6).

**Figure 7:**
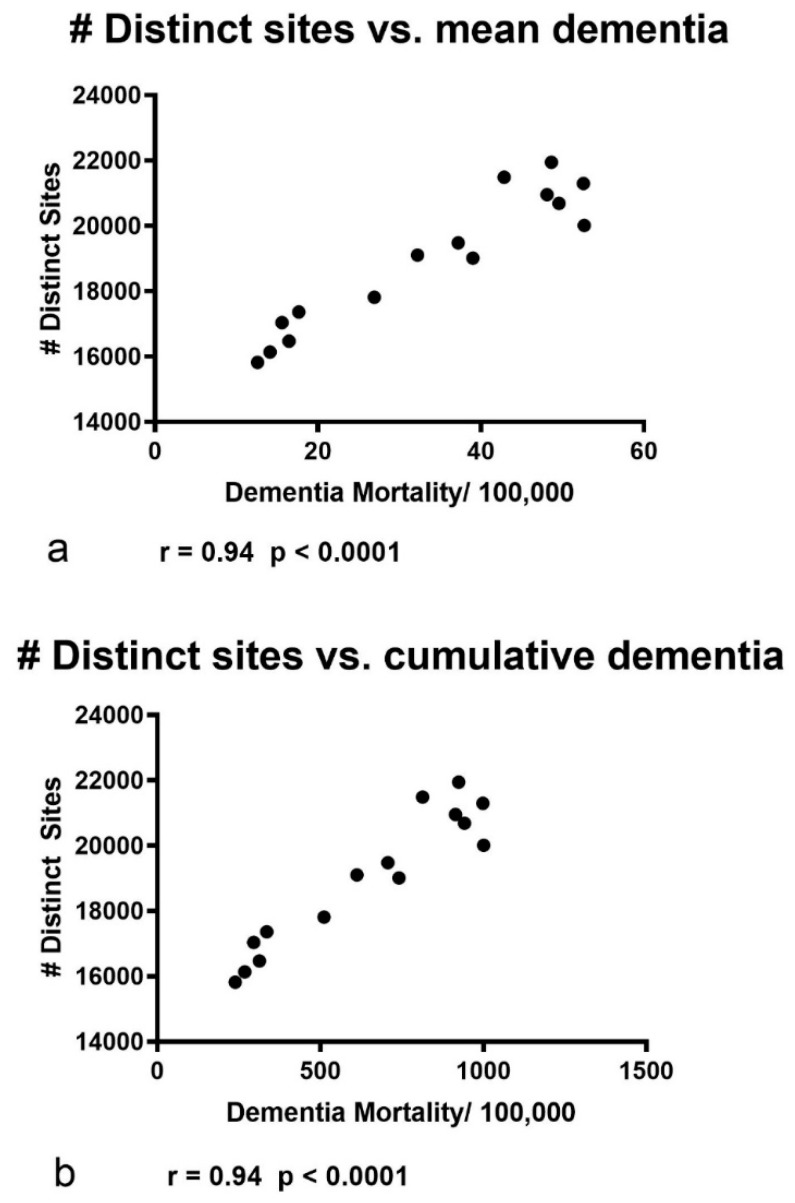
Pearson’s r correlation analyses of mean and cumulative vascular and unspecified dementia mortality versus the number of distinct mining sites. (**a**) A significant correlation was found between the number of distinct sites per year vs. the mean age-adjusted vascular and unspecified dementia mortality/100,000 population per year (r = 0.94; *p* < 0.0001; xy pairs = 15; data depicted in Figure 6). (**b**) A similar analysis comparing the total number of distinct sites to the cumulative age-adjusted dementia mortality/100,000 population per year yielded identical results (r = 0.94; *p* < 0.001; xy pairs = 15).

When a Pearson’s r correlation analysis of the data, depicted in Figure 6, was used to compare the two data sets, a significant positive correlation was found between the number of distinct sites per year vs. the *mean* age-adjusted dementia/100,000 population/year (Figure 7a; r = 0.946; *p* < 0.0001; 15 xy pairs). A similar analysis comparing the number of distinct sites to the *cumulative* age-adjusted dementia/100,000 population per year yielded identical results (Figure 7b; r = 0.946; *p* < 0.001; 15 xy pairs). 

**Figure 8:**
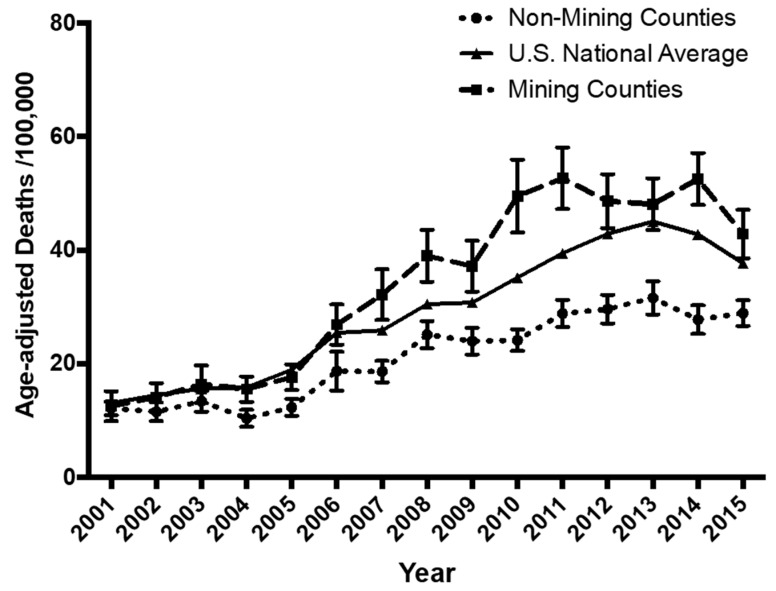
Comparison of vascular and unspecified dementia mortalities for West Virginia MTM and non-mining counties with the United States’ national average. These data suggest that while the rates for all three populations are initially the same, the data diverge around 2007. After 2007, WV MTM counties exhibited more steeply increasing rates of dementia mortality, than did non-mining West Virginia counties or the U.S. on average. Comparison of post-2007 data for non-MTM WV counties with that for the U.S. as a whole suggests that living in the former may actually confer a degree of protection relative to the county as a whole. This remains to be explored. National average data (means and Standard Errors of the Mean (S.E.M.s) only) were downloaded from the Center for Disease Control Wonder website, https://wonder.cdc.gov/, and the error bars are within the scale of the symbol.

Comparison of the age-adjusted dementia mortalities/100,000 population for West Virginia MTM and non-mining counties with the United States’ national average suggests that while the rates for all three populations are initially the same, the data diverge around 2007 (Figure 8). After 2007, WV MTM-counties had increasingly greater dementia mortality than either non-mining West Virginia counties or the U.S. as a whole. We note that rates of dementia mortality in non-mining WV counties were lower than that seen for the U.S. as a whole.

**Figure 9:**
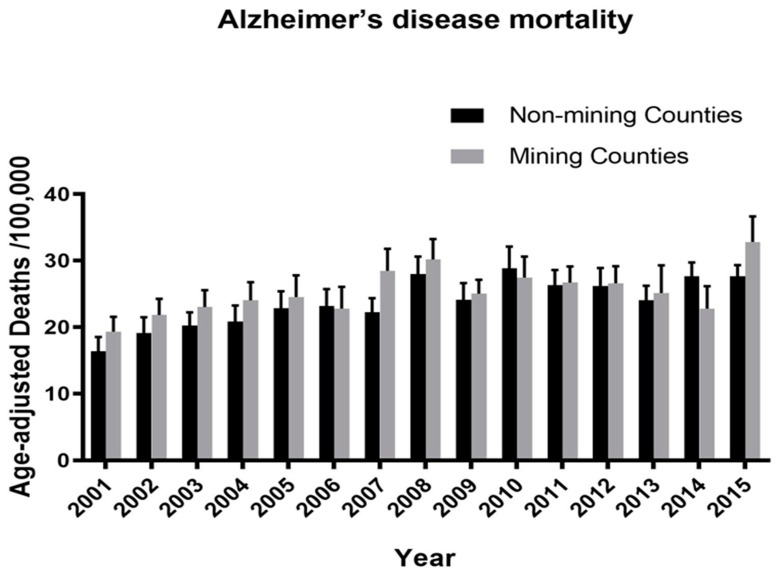
Alzheimer’s disease mortality in MTM vs. non-mining counties in West Virginia over the years spanning 2000–2015. The analysis did not find an effect of MTM on age-adjusted Alzheimer’s disease mortality/100,000 population (F = 0.38; df = 1, 52; *p* = 0.53). Likewise, there was no interaction between year and mining (F = 0.70; df = 14; 728; *p* = 0.76). However, similar to the finding for dementia mortality, a significant effect of year (F = 4.34; df = 14, 728; *p* < 0.0001) was found.

*Alzheimer’s disease findings (*Figure 9*).* A 2-way repeated measures ANOVA was used to compare age-adjusted mortality/100,000 population from Alzheimer’s disease in MTM vs. non-MTM counties for the years inclusive of 2001–2015. The analysis did *not* find an effect of residing in an MTM county on age-adjusted Alzheimer’s disease (AD) mortality/100,000 population (Figure 9; F = 0.38; df = 1, 52; *p* = 0.53), i.e., living in a mining county per se was not associated with increased AD over the 15 year period. Likewise, there was no selective interaction between year and county mining status in MTM vs. non-MTM counties (F = 0.70; df = 14, 728; *p* = 0.76), i.e., the chances of dying from AD in any particular year were roughly the same for MTM and non-MTM counties. However, similar to the finding for unspecified and vascular dementia mortality, a significant effect of year (F = 4.34; df = 14, 728; *p* < 0.0001) was found, meaning there was a difference (increase) in Alzheimer’s disease mortality over the 15 year period which occurred roughly equally in *both* MTM and non-MTM counties.

**Figure 10:**
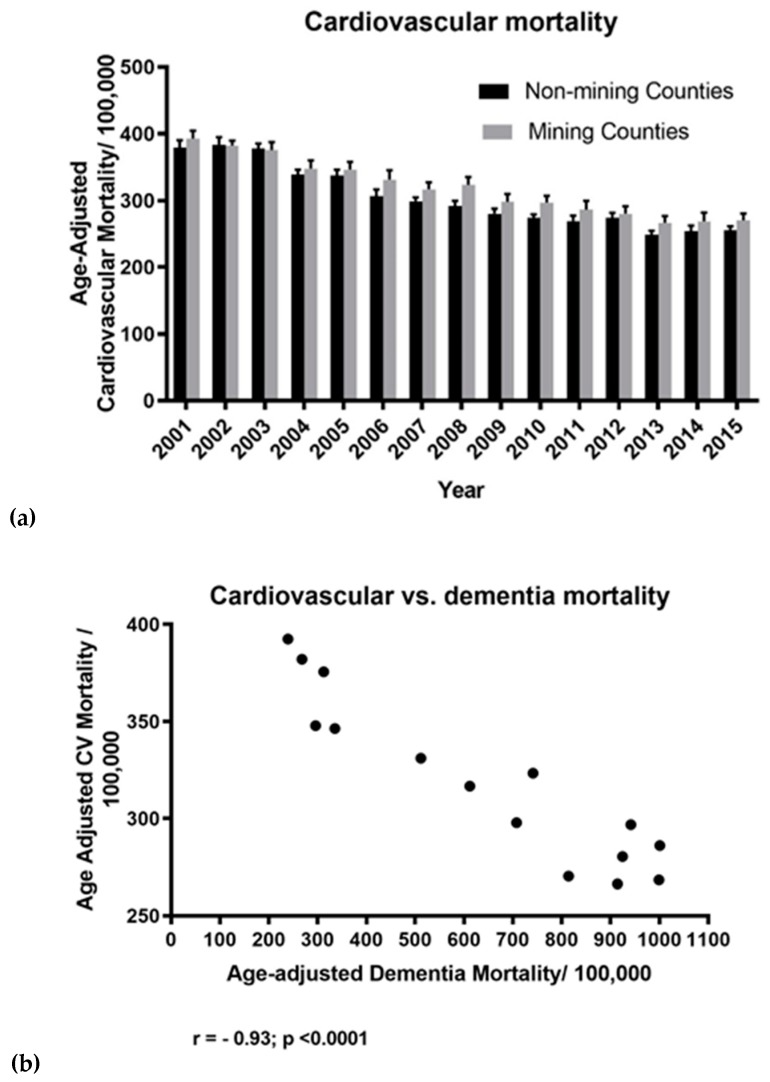
Cardiovascular disease in MTM vs. non-mining counties in West Virginia over the years spanning 2000–2015. (**a**) A two-factor repeated measures ANOVA showed a significant difference (decline) in cardiovascular deaths over this period (F = 63.25; df = 14, 714; *p* < 0.0001). Although a possible trend was evident, there was no significant effect of MTM when analyzed over the 15 year period (F = 2.84; df = 1, 51; *p* = 0.097). Likewise, there was no interaction of year with mining (F = 0.64; df = 14, 714; *p* = 0.82). (**b**) A Pearson’s r product-moment correlation analysis (mining counties alone) over the 15 year period revealed a significant negative correlation between cardiovascular mortality/100,000 per year vs. the cumulative age-adjusted dementia mortality/100,000 per year (r = −0.93; *p* < 0.0001; 15 xy pairs).

*Cardiovascular disease* vs. *dementia:* A two-factor repeated measures ANOVA examination of major cardiovascular disease mortality in MTM counties for the years 2001–2015 was undertaken to determine if this might be a confounding factor with respect to the dementia vs. MTM findings. This showed a significant difference (decline) in cardiovascular disease (CVD)-related deaths over this period (Figure 10a; F = 63.25; df = 14, 714; *p* < 0.0001). Although a possible trend was evident, there was no significant effect of MTM on CVD mortality when analyzed over the 15 year period (F = 2.84; df = 1, 51; *p* = 0.097). There was no interaction of year and county mining status on CVD mortality (F = 0.64; df = 14, 714; *p* = 0.82). A Pearson’s r correlation analysis was used to assess a possible relationship between CVD vs. dementia mortalities for the years 2001–2015. A significant negative correlation was found between CVD mortality/100,000 population vs. the mean age-adjusted dementia mortality/100,000 population per year (Figure 10b; r = −0.935; *p* < 0.0001; 15 xy pairs).

*Socioeconomic factors* vs. *dementia mortality.* We also endeavored to determine if socioeconomic factors could explain the disparities in dementia mortality in MTM vs. non-MTM counties. Data were averaged over the 2005–2009 plus 2010–2014 time periods (again, this was the longest period of homogeneously organized data available that fit within our original 15 year timeframe for MTM and dementia data). Unpaired t-tests were used to compare the percentage of individuals aged 24 or older with educational attainment limited to a high school diploma (or equivalent) in MTM vs. non-MTM counties. No significant differences were found on this measure (t = 1.53; df = 53; *p* = 0.13; Means ± S.E.M.s = 45.43 ± 0.96%; 43.36 ± 0.84% respectively). Likewise, comparison of the percentage of individuals in MTM vs. non-MTM counties aged 24 or older with a bachelor’s degree or higher found no differences on this measure. (t = 0.93; df = 53; *p* = 0.35; Means = S.E.M.s = 13.59 ± 1.30%; 15.12 ± 0.96% respectively). With respect to economic factors vs. dementia mortality, an unpaired t-test also found no differences in mean household incomes in the MTM vs. non-MTM counties (t = 0.0.87; df = 52; *p* = 0.38; Means ± S.E.M.s = $47,724 ± $1717.00; $49,620 ± $1290.00 respectively).

A multivariate analysis of covariance (ANCOVA) was then conducted with SAS software (version 9.4; SAS Institute Inc., Cary, NC, USA) to determine the effect of county mining status on dementia mortality in the absence of education and income factors. Since the outcomes of the educational attainment comparisons described above were roughly the same we chose to use the high school diploma or equivalent data for this variable. After controlling for socioeconomic factors, the dementia mortality rate was 15.60 (±3.14 S.E.M.; 95% C.I.: 9.28–21.91) times greater in MTM counties as compared to non-MTM counties (Table 2; *p* < 0.0001).

**Figure 11:**
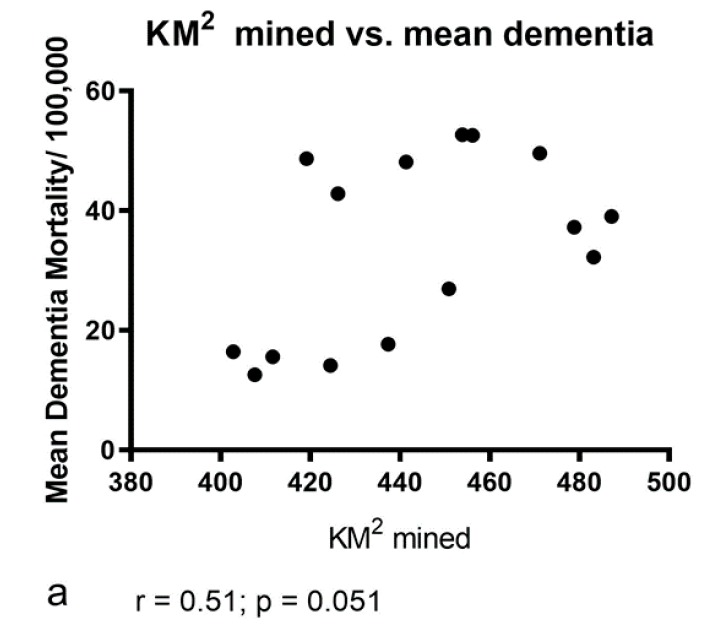

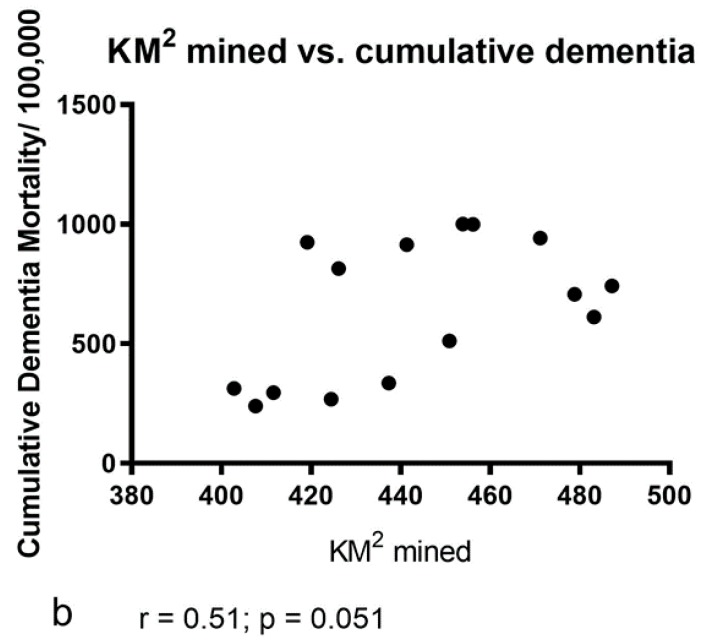
(**a**) No significant correlation was found between the extent of mining (km^2^) vs. the mean number of age-adjusted deaths from dementia/100,000 population (r = 0.51; *p* = 0.051; 15 xy pairs). (**b**) No significant correlation was found between the extent of mining (km^2^) vs. the cumulative number of age-adjusted deaths from dementia/100,000 population (: r = 0.51; *p* = 0.051; 15 xy pairs).

*Km^2^ mined* vs. *dementia mortality (*Figure 11). Although the highest rates of dementia were seen in counties with the highest rates of mining (e.g., Boone, Kanawha), only marginally non-significant statistical correlations were found between the number of km^2^ mined and the rates of age-adjusted dementia mortality/100,000 population (Two-tailed Pearson r correlation analysis; Figure 11a,b; both r = 0.51; *p* = 0.051; 15 xy pairs).

**Figure 12:**
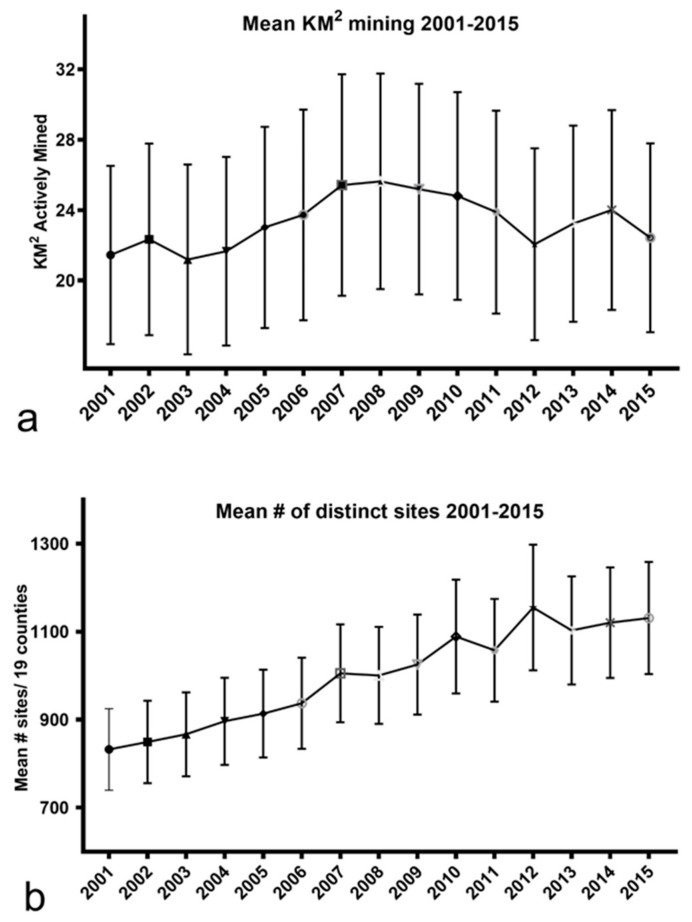
Mean *km^2^* mined vs. mean *distinct sites* mined per year from 2001–2015. (**a**) Km^2^ mined remained relatively flat over the 15 year period whereas (**b**) the number of distinct sites rose steadily. Error bars = S.E.M.s.

Comparing the yearly km^2^ mined per year vs. the number of distinct sites mined per year shows that the former remained relatively flat over the 15 years whereas the latter steadily increased (Figure 12).

## 4. Discussion

To summarize the main findings, a highly significant increase in the age-adjusted vascular or unspecified dementia mortality/100,000 population was found in West Virginian counties where MTM occurred from 2001–2015, relative to that of non-MTM counties over the same period. The mean, peak and cumulative rates of dementia across the 19 counties were all significantly elevated in MTM-counties. Of particular import, there was a strong correlation between the number of distinct mining sites per county vs. the rate of dementia mortality for that county, in contrast to the marginally non-significant association found between the actual km^2^ mined vs. the rate of dementia mortality. In addition, we saw a significant increase in the rates of both unspecified and Alzheimer’s dementia mortality over these 15 years in both MTM and non-MTM counties. This is consistent with reports of elevated all-type dementia and neurological diseases in the U.S., other industrialized countries and the developing world over this period [40,41].

“Ecological” studies such as this are widely appreciated for generating hypotheses concerning environmental health effects [32]. An inherent limitation to this work is that deidentified death certificate data cannot be used to draw conclusions about particular individuals, especially in the absence of clearly identified intervening variables (see [42] for a cogent overview of this issue). Follow-up cohort and/or case-controlled studies are now needed to conclude whether, and to what degree the inhalation of mining dust from MTM operations (or exposure to other detrimental MTM sequelae) causes increased dementia mortality in the mining counties. Below we list a number of key areas for further investigation that would benefit from targeted federal and state funding.

*Determination of individual PM dosage levels in relation to dementia mortality.* While data acquired near MTM sites indicate regional increases in PM concentrations, detailed data pertaining to individual respiratory dosage levels must now be obtained. Areas exposed to PM fallout from MTM are differentially affected by the topography of surrounding hills and valleys. These, in turn, are uniquely impacted by season, wind speeds and direction [10]. One might expect an individual living east of a mining operation to receive more exposure to wind borne particulates than one living equidistant to the west, due to prevailing easterly winds. Ongoing, and on-site, measurements of air quality are required to establish actual individual exposures to PM in any location. Such measures have, in fact, been specifically called for in a recent review of the existing literature pertaining to the effects of MTM on human health [5]. Targeted studies to determine individual levels of PM exposure in relation to individual hospital admissions and incidence of dementia diagnoses must now follow, as has been done for other illnesses [43]. New technology to assess particulate matter concentrations from satellite images is now available [17] and this could prove useful for a relatively continuous assessment of air quality in localized areas.

It must be noted that death certificates indicate the location where death occurred, they do not reveal how long individuals lived in a particular county and whether they had additional exposures to PM. As also discussed by Esch and Hendryx [2], here we have called the counties of central Appalachia where the most surface mining occurs and where most surface mining is increasingly of the MTM type, “MTM counties”. It must be acknowledged that underground and other mining is extensive in some “non-MTM counties”. Thus, the differences between MTM counties and those we have designated as non-MTM counties are likely not absolute but rather a matter of degree. And this may be reflected in the observation that, taken individually, a few MTM counties had very low dementia mortality and some non-MTM counties had relatively high dementia mortality. Although, the majority of the 19 MTM counties had dementia mortality rankings which placed them in the top 25 of all WV counties, there were some notable exceptions to this including Mason (30th), Raleigh (27th), Mingo (46th) and Pocahontas (54th) counties, which had less dementia mortality than many non-MTM counties. Likewise, counties such as Upshur (7th), Marion (8th) and Ohio (13th), with modest or no surface mining over the period of study, ranked relatively high in dementia mortality, no doubt underscoring the role of additional factors in dementia mortality. The actual amount of mining-generated PM exposure received by individuals living in MTM *and* perhaps also some non-MTM counties awaits assessment, in relation to individual de-identified health outcomes, e.g., as in [13].

*Determination of a premorbidity period.* If indeed inhalation of MTM PM is a factor in the etiology of dementia and other neurodegenerative diseases, the existence and nature of the pre-morbidity period becomes of interest. We note that surface mining in many of the counties studied here has been ongoing since the 1970s. For dementia in general, the presence of a “preclinical period” when pathophysiologic processes have begun, but during which time significant cognitive decline is not yet evident, is now recognized [44]. This is estimated to be a decade or more in some individuals [44]. In the course of this work, we did sometimes, *but not consistently*, observe a seemingly temporal relationship between increased mining and ensuing upswings in dementia mortality and that the lag time between the former and latter was on the order of several years. This observation demands follow-up, as if exposure to airborne nanoparticulates ultimately proves to be an etiological factor for dementia, the rate at which neurological changes begin to manifest post-exposure becomes critical. Work with both humans and animals shows that exposure to components of air pollution leads to brain inflammation and the expression of molecular markers of neuroinflammation, Alzheimer’s Disease and dementia [25,45,46]. Under laboratory conditions, some responses occur quite rapidly: exposure of mice to nanoparticles (NPs), in concentrations which left them otherwise healthy, resulted in elevated brain oxidative stress markers within 45 hours of exposure [22]. Whether and how similar neurodegenerative processes would develop in humans after exposure to NPs from MTM is unknown and this calls for focused data collection. Again, more study of individual exposures, followed by collection of detailed health histories is now needed. Post-mortem histological examination of donated brain specimens would further help determine disease progression parameters in individuals living near MTM operations.

We note Figure 8 suggests that living in a non-MTM West Virginia county confers a protective effect against non-Alzheimer’s dementia mortality relative to the U.S. as a whole. This finding is difficult to explain without a further nationwide comparison of key lifestyle and socioeconomic factors associated with increased risk for dementia and cognitive decline in general. However, of interest is a recent report [47] that an “enriched environment” effect accrues to the brains of those living near heavily forested areas: despite decades of surface mining, West Virginia remains the third most forested state in the U.S. with approximately 79% coverage [48].

Enriched environments, like higher levels of education, have been linked to a greater complexity of brain structure and function, in turn thought to be related to the increased “cognitive reserve” associated with elevated protection against dementia and AD [37].

*Analyses of Alzheimer’s Disease* vs. *MTM.* Although rates of vascular or unspecified dementia mortality were significantly elevated in MTM counties, there was no similar finding for Alzheimer’s disease. Why? This study spanned only one edition of the ICD, precluding inconsistencies across the years due to changing classifications on death certificates [33]. A number of factors may be at play. We did find a significant increase in all types of dementia over the 2001–2015 timeframe, which is consistent with national and international trends [40,41]. One reason for the increase in all dementia diagnoses is that people are now living longer, i.e., they are not dying of other causes such as cardiovascular disease and stroke first [40]. In addition, some of the increase in all diagnoses may be attributable to a general increase in awareness and recognition of dementia and AD. A designation of death from AD includes ICD codes G30-G30.9. It features loss of short-term memory, confusion, difficulty thinking and changes in language, behavior and personality. A diagnosis of AD in the living is often described as one of exclusion after all other causes of dementia have been ruled out. The more general diagnoses of vascular dementia (ICD codes F01) or unspecified dementia (F03) are based on problems with at least two or more brain functions, such as memory and language. Despite vigorous efforts to discover blood borne factors indicative of AD, at the time of writing no definitive blood test is widely available for AD. Brain imaging, increasingly useful for revealing the cortical atrophy or the amyloid deposits and neurofibrillary tangles specific to AD [49], is still prohibitively expensive for many. Thus, the posthumous demonstration of amyloid plaques and neurofibrillary tangles in autopsied brain tissues remains the gold standard for an AD diagnosis, yet this procedure is relatively rare following death from dementia. Internists called to a recently deceased’s bedside to fill out a death certificate, sometimes without having known the deceased in life, may err on the side of the more generalized diagnosis of dementia. As a result of these factors, many suspect the actual rate of AD is under-represented on death certificates [40]. Given these uncertainties, it would be premature to conclude that dementia of the Alzheimer’s type is unaffected by those conditions surrounding the increase in unspecified and vascular dementia mortality seen here. Given this, our comments (below) concerning the need for further studies are intended to apply equally to all types of dementia.

*Cardiovascular and socioeconomic factors.* In contrast to that seen for all dementia mortality, examination of all-cause cardiovascular disease (CVD) mortality data for the period spanning 2001–2015 showed cardiovascular mortality, in fact, steadily declined over the 15 year period studied. Perhaps this reflects ongoing and vigorous public health efforts to decrease these statistics in West Virginia. Whatever the cause, there was an inverse relationship seen between dementia mortality and CVD mortality. One explanation is that the population may now be escaping CVD only to be exposed to a greater risk for dementia conferred by advancing age. This remains to be established however. Certainly, with respect to increased CVD mortality in MTM counties, a strong association between CVD and dementia mortality has been reported previously in a much larger study specifically focused on the matter [2]. In the current, smaller, study we saw a trend in this direction including a p-value of 0.09 and elevated MTM county CVD mortality in 14 of 15 years examined (Figure 10). At present we posit that the limited number of counties (55) studied here resulted in less statistical power to establish effects of MTM on CVD mortality than that previous work [2]. We currently conclude that the finding of increased CVD mortality in MTM counties still holds.

As for socioeconomic factors, these have been linked to rates of dementia under other circumstances [37,38]. Here, when directly compared in the absence of other variables, there was no significant differences in mean income levels for MTM vs. non-MTM counties, although a relationship between income and dementia was seen as a nuisance variable in the ANCOVA. We conclude that while a frequent observation has been that hazardous environmental sites tend to be located in lower income areas [50] this may not hold for WV MTM counties. An explanation may lie in the fact that mining jobs are relatively well-compensated: ~$49,000/year on average (https://www.bls.gov/opub/ted/2018/employment-and-wages-in-mining-industries.htm), placing nine of the 19 mining counties in the top half of all West Virginia counties in per capita income from 2011–2015 (2011–2015 ACS 5 year estimate). Likewise, there were no differences found between educational attainment limited to a high school diploma or equivalent, or the percent of individuals 24 years or older with a bachelor’s degree or higher in non-mining vs. mining counties. Readers who are interested in further demographic information for the state may find it at https://datausa.io/profile/geo/west-virginia/.

*Other risk factors.* If any, the degree to which diabetes, obesity, cancer, hypertension and a host of poor lifestyle choices plays a role in the current dementia statistics, remains to be elucidated in future work [51]. There may be yet residual confounds stemming from lack of equal access to healthcare and other services, exercise, diet and community support, to name but a few unaddressed issues. The goal of the present study therefore remains one of determining an urgent need for further investigation. Fully evaluating the impact of all of these factors on dementia mortality in WV awaits a well-funded team of epidemiologists.

## 5. Conclusions

The mining industry, and the U.S. Department of the Interior under the Trump administration, argued in their 2017 announcement of funding withdrawal from the study of MTM-related health effects commissioned by the National Academies of Science, Engineering and Medicine, that further study is unwarranted because there is now less mining [6,7]. Our data not only call into question the assertion of less mining (Figure 12a) it also indicates only a weak association between the number of km^2^ mined and the rate of dementia mortality (Figure 11). Instead, it was the *number of distinct mining sites* that strongly and positively correlated with the dementia mortality rate (Figure 7). The reasons for this are not clear. One possibility is that once individuals are initially exposed to some, as of yet undefined, baseline amount or type of PM, the triggering of pathophysiological processes may ensue irrespective of the amount of mining-related PM exposure that follows. And, because there are both widely dispersed, and high numbers of, MTM sites in some counties, (see Figure 2, Table 1 and [39], PM exposure per se may be independent of the number of km^2^ ultimately mined. This interpretation is supported by our observation that counties with seemingly little mining often had dementia rates that were on par with counties with many more km^2^ of mining. We now know that brain inflammation, once initiated, may continue relentlessly thereafter [52,53]. For those already exposed, immediate reductions in the number of acres mined may not impact negative health sequelae previously set in motion and it is possible that ill-health effects are yet to manifest in some areas and populations. In addition to the proposed “preclinical period” of approximately 10 years [44], other data indicate survival after a diagnosis of dementia varies from 3 to 12 years [54]. Hence, a sobering conclusion from the present findings is that (no matter the cause) elevated dementia mortality in these WV counties may be present for the next 10–15 years. Public health resources may need to be selectively allocated toward MTM counties where the future caregiver burden and public health cost challenges of dementia could prove to be higher than in other parts of the state. Confirming the current observations with rigorous epidemiological approaches now becomes imperative.

## Figures and Tables

**Table 1:** Mining site data and cumulative dementia deaths 2001–2015 by county.

County	Minimum Distinct Sites	Maximum Distinct Sites	Mean Distinct Sites	Cumulative Distinct Sites	Cumulative Dementia Deaths/100,000
Boone	1364	1556	1481	22,221	884.8
Cabell	277	390	335	5024	642.6
Clay	590	792	686	11,027	566.3
Fayette	1109	1677	1418	21,272	546.3
Greenbrier	1238	1978	1577	23,654	409.8
Kanawha	1393	1968	1696	25,437	711.2
Lincoln	725	970	854	12,810	788.5
Logan	1124	1237	1179	17,682	364.6
Mason	250	357	309	4637	320.6
McDowell	147	1236	1031	15,461	385.6
Mercer	338	486	417	6251	655.8
Mingo	1142	1321	1260	18,897	227.8
Nicholas	1135	1687	1426	21,396	438.3
Pocahontas	1288	1858	1562	23,430	179.7
Putnam	176	2058	358	5372	705.0
Raleigh	772	2579	1036	15,544	345.1
Summers	422	602	521	7823	435.8
Wayne	712	1633	879	13,189	496.1
Wyoming	785	1118	954	14,315	514.1
Totals	14,987	25,503	18,979	285,415	9618.00

**Table 2:** ANCOVA Results. Effects of county MTM status after controlling for socioeconomic factors 2005–2015.

Effect	Estimate	Standard Error	DF	t Value	Pr > |t|	Alpha	Lower	Upper
Intercept	−26.75	37.71	49	−0.71	0.481	0.05	−102.55	49.02
Mining	15.60	3.14	49	4.96	<0.0001	0.05	9.28	21.91
Non−Mining	0							
Hs	−0.031	0.39	49	−0.08	0.93	0.05	−0.83	0.77
Income	0.0007	0.0003	49	2.31	0.025	0.05	0.0001	0.0014
Poverty	0.93	0.56	49	1.64	0.107	0.05	−0.21	2.0759

*Hs* refers to the average percentage of individuals aged 24 or older per county having attained a high school diploma or equivalent for the years 2005–2014. *Income* refers to the mean household income/county for 2005–2014. *Poverty* refers to the percentage of households that fell below the U.S. Census poverty threshold for 2005–2014 (see *Methods* for definition of poverty threshold).
